# High-Level Expression, Purification and Characterization of a Constitutively Active Thromboxane A2 Receptor Polymorphic Variant

**DOI:** 10.1371/journal.pone.0076481

**Published:** 2013-09-23

**Authors:** Bing Xu, Raja Chakraborty, Markus Eilers, Shyamala Dakshinamurti, Joe D. O’Neil, Steven O. Smith, Rajinder P. Bhullar, Prashen Chelikani

**Affiliations:** 1 Department of Oral Biology, University of Manitoba, Winnipeg, Manitoba, Canada; 2 Department of Biochemistry and Cell Biology, Stony Brook University, Stony Brook, New York, United States of America; 3 Department of Physiology, Pediatrics, University of Manitoba, Winnipeg, Manitoba, Canada; 4 Manitoba Institute of Child Health, Winnipeg, Manitoba, Canada; 5 Department of Chemistry, University of Manitoba, Winnipeg, Manitoba, Canada; Southern Illinois University School of Medicine, United States of America

## Abstract

G protein-coupled receptors (GPCRs) exhibit some level of basal signaling even in the absence of a bound agonist. This basal or constitutive signaling can have important pathophysiological roles. In the past few years, a number of high resolution crystal structures of GPCRs have been reported, including two crystal structures of constitutively active mutants (CAM) of the dim-light receptor, rhodopsin. The structural characterizations of CAMs are impeded by the lack of proper expression systems. The thromboxane A2 receptor (TP) is a GPCR that mediates vasoconstriction and promotes thrombosis in response to the binding of thromboxane. Here, we report on the expression and purification of a genetic variant and CAM in TP, namely A160T, using tetracycline-inducible HEK293S-TetR and HEK293S (GnTI¯)-TetR cell lines. Expression of the TP and the A160T genes in these mammalian cell lines resulted in a 4-fold increase in expression to a level of 15.8 ±0.3 pmol of receptor/mg of membrane protein. The receptors expressed in the HEK293S (GnTI^-^)-TetR cell line showed homogeneous glycosylation. The functional yield of the receptors using a single step affinity purification was 45 µg/10^6^ cells. Temperature- dependent secondary structure changes of the purified TP and A160T receptors were characterized using circular dichroism (CD) spectropolarimetry. The CD spectra shows that the loss of activity or thermal sensitivity that was previously observed for the A160T mutant, is not owing to large unfolding of the protein but rather to a more subtle effect. This is the first study to report on the successful high-level expression, purification, and biophysical characterization of a naturally occurring, diffusible ligand activated GPCR CAM.

## Introduction

G protein-coupled receptors (GPCRs) comprise the largest family of membrane proteins encoded by the human genome. On binding to extracellular stimuli, these receptors activate intracellular proteins thereby providing an important link between the cell and its environment [1]. A substantial number of GPCRs in humans harbor genetic variants [2] including nucleotide insertion or deletion, as well as single nucleotide changes referred to as single nucleotide polymorphisms (SNPs). Some of these SNPs lock the GPCR in an active form, and initiate intracellular signaling even in the absence of extracellular stimuli, these are referred to as constitutively active mutants (CAMs). The structural characterization of these CAMs is impeded by the lack of proper expression systems, as most often high-level expression of these CAMs appear to be toxic to the cells [3]. An approach to circumvent this hurdle is the use of a tetracycline-inducible HEK293 cell line [4]. Recently the structures of two CAM GPCRs were reported (PDB ID: 2X72 and 4A4M) using this cell line, although the CAMs required stabilization using an engineered disulfide bond [5,6].

The human thromboxane A2 receptor (TP) belongs to the prostanoid subfamily of GPCRs. The receptor mediates vasoconstriction and thrombosis on binding to thromboxane (TXA2) thereby playing an important role in cardiovascular disease and stroke [7]. TP was first cloned in 1991 and shown to exist in two isoforms in humans, TPα and TPβ, differing only in their C-terminus [8]. Recently, we reported the first CAM in TPα (henceforth referred to as TP or WT-TP), the genetic variant A160T present in transmembrane (TM) helix 4 [9]. Though the clinical relevance of this CAM in TP is yet to be elucidated, based on CAMs at similar positions in rhodopsin that lead to retinitis pigmentosa, it is likely A160T mutation causes cardiovascular disease progression.

A high-resolution structure of a prostanoid receptor has not been determined. Recently, glycosylated human TP was expressed in Sf-9 cells using an optimized baculovirus expression system [10]. From heterologous expression in HEK293 cells, TP protein levels of 0.5-2.0 pmol/mg of membrane protein have been reported [11,12]. The main goal of the present work was to improve the expression levels of both the TP and CAMs for high-resolution structural studies. Towards this aim, codon-optimized TP and the A160T mutant were synthesized, and transiently expressed in both COS-1 and HEK293 cells. Expression of these constructs resulted in yields of 3.8 ±0.3 picomoles of WT-TP and 1.8 ±0.4 picomoles of A160T per milligram of membrane protein, respectively. Next, expression of these genes in HEK293S-TetR cells resulted in a 4-fold increase in expression, resulting in yields of 15.8 ±0.3 pmol of receptor/mg of membrane protein. To date, this expression level is the highest reported for any diffusible ligand activated GPCR CAM. The WT-TP and the A160T mutant expressed in the HEK293S (GnTI¯)-TetR cell line showed homogenous and restricted N-glycosylation. Secondary structure analysis of the purified receptors was pursued by circular dichroism (CD) spectropolarimetry.

## Results and Discussion

### Expression of TP and the A160T CAM in HEK293S-TetR and HEK293S-TetR (GnTI^-^) cell lines

**Figure 1:**
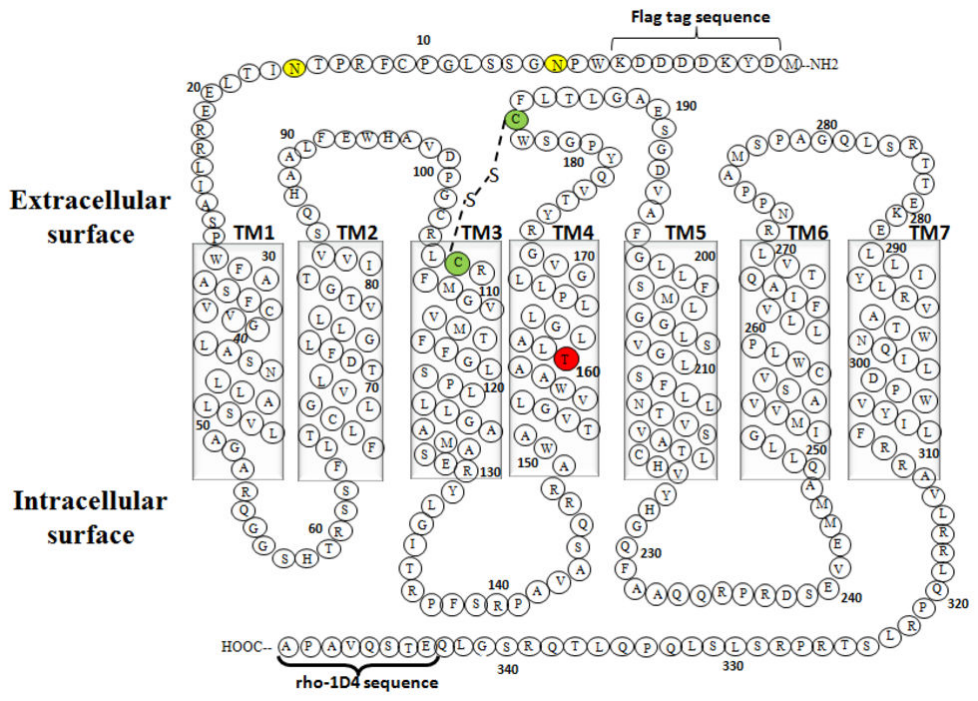
Secondary structure representation of the TPα amino acid sequence with the genetic variant A160T. Amino acids are shown in single lettered codes, and the residue numbering excludes the epitope tags at both ends. Shown are the seven transmembrane helices (TM1-7), the FLAG sequence at the N-terminus, the N-glycosylated residues Asn4 and Asn16 (yellow colored residues), the disulphide bond between Cys 105 and Cys183 (green colored residues), and the rho-1D4 octapeptide epitope tag at the C-terminus. The genetic variant A160T (residue 4.53 using Ballesteros-Weinstein numbering) on TM4 is highlighted in red.

**Figure 2:**
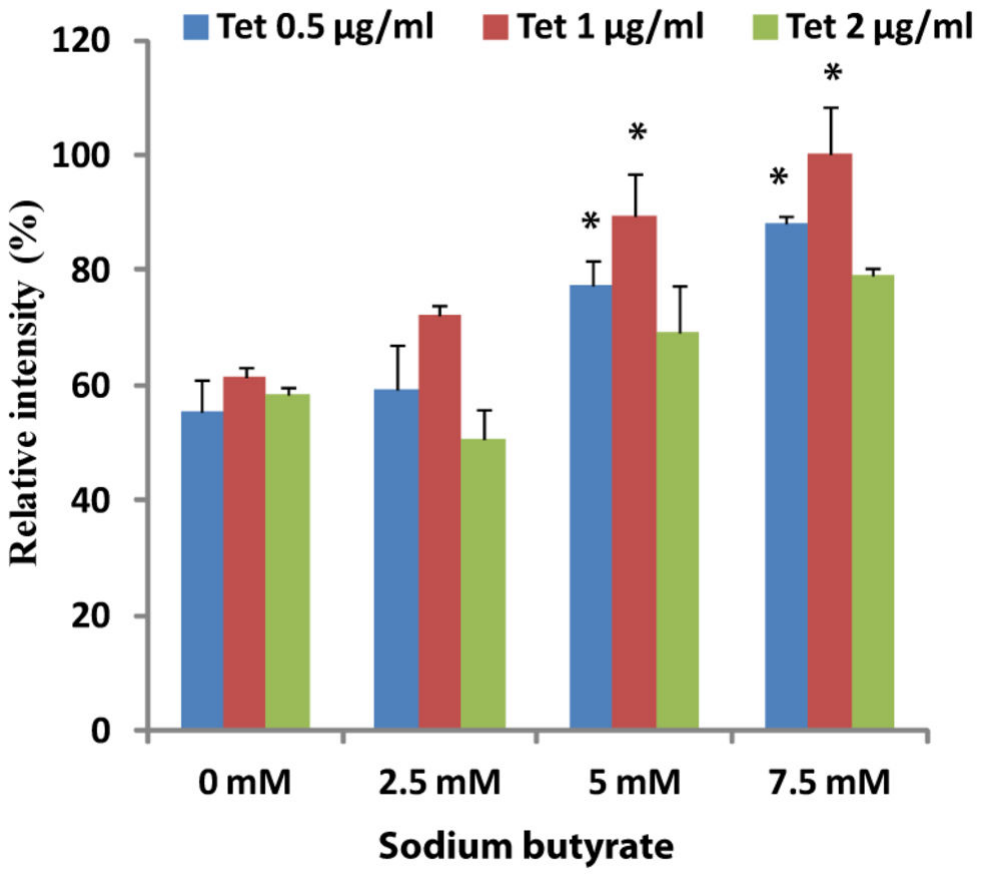
Optimization of TP expression in HEK293S-TetR inducible cells. The expression of TP in the HEK293S-TetR was induced by tetracycline and/or sodium butyrate as shown. Following induction, the samples were harvested, solubilized, and analyzed using the dot blot technique and probed with FLAG antibody. The results were quantified using spot densitometry (ImageJ software) and normalized to 100% of relative intensity. A one way ANOVA with tukey’s post hoc test was done where the samples obtained from 5 and 7.5 mM of sodium butyrate with 0.5 and 1 µg of Tet/ml showed statistical significance at *p<0.05 compared to 0 mM sodium butyrate and 0.5 and 1 µg of Tet/ml.

**Figure 3:**
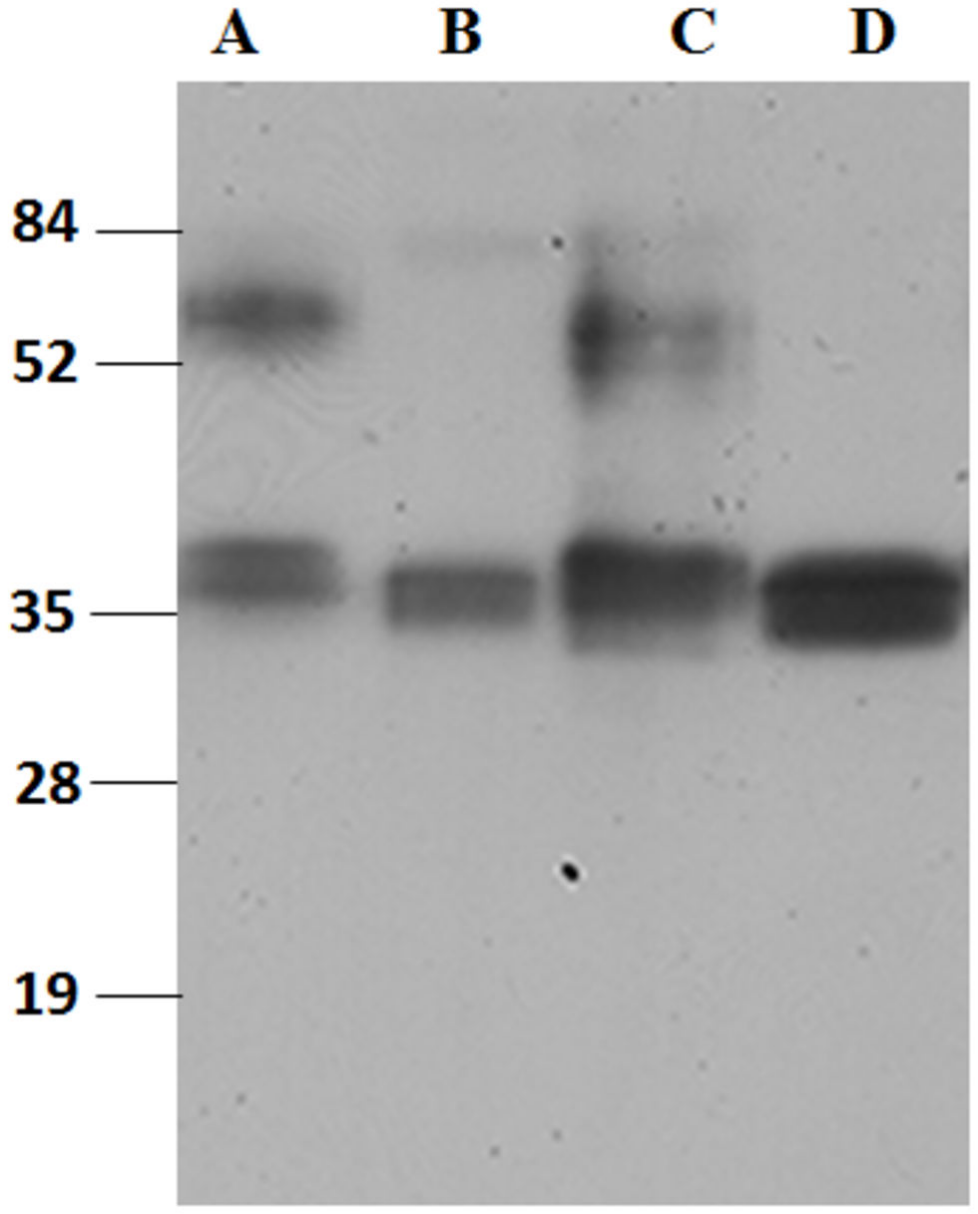
Immunoblot analysis of TP and A160T using the monoclonal FLAG antibody. TP and the A160T variant expressed in HEK293S-TetR stable cell line (lanes A and C). By using a HEK293S (GnTI^-^) cell line defective in N-acetylglucosamine transferase I, TP and A160T were expressed with restricted and homogeneous N-glycosylation (lanes B and D). 5 µl of protein were loaded into all wells, and western blot analysis carried out using the FLAG antibody. Mobility of molecular weight standards is indicated next to the gel. The epitope tag for the monoclonal FLAG antibody was added to the N-terminus of the TP receptor.

The WT-TP and A160T genes that were synthesized and used in the current study had the same salient features as those previously described for the β_2_-AR gene [13]. In addition to simplify detection of the full-length protein and purification, a FLAG-epitope tag (DYKDDDDK) and rho-1D4 octapeptide (ETSQVAPA) tag were added to the N-terminus and C-terminus, respectively (Figure **1**). Transient expression of these genes in either COS-1 or HEK293S cells resulted in expression levels of 3.8 ±0.3 pmol TP/mg and 1.8 ±0.4 pmol A160T/mg of membrane protein [9,14]. To increase the expression levels, construction of stable cell lines using the HEK293S-TetR inducible system was explored. The expression was optimized by varying the concentrations of the inducers, both tetracycline and sodium butyrate, and the results quantified by western blotting and spot densitometry. The addition of sodium butyrate and tetracycline had a cumulative effect, with 7.5mM sodium butyrate found to be the optimum (Figure **2**). Interestingly, tetracycline alone was able to induce up to 60%, of the level of expression of the WT-TP. However, after induction with both tetracycline and sodium butyrate, WT-TP and A160T were expressed at 15.8 ±0.3 pmol/mg and 2.1 ±0.3 pmol/mg of membrane protein, respectively, as determined by radiolabeled antagonist [^3^H] SQ 29,548 binding. The expression level determined from the radioligand assay for the A160T CAM is not a true indicator of its expression. This is because the radioligand used ([^3^H] SQ 29,548) is an antagonist for TP, and CAMs being in an active state have low affinity for antagonists. Active state stabilizing mutations of the A2A adenosine receptor used for crystallization showed greatly reduced binding of five antagonists [15]. Indeed, based on intensity of the immunoblots (Figure **3**) and functional yield obtained after purification, both the WT-TP and A160T CAM are expressed at similar levels (please see purification section).

Immunoblot analysis showed the WT-TP and A160T mutant expressed in HEK293S-TetR stable cell lines consist of two major bands with molecular masses in the range of 30-55 kDa (Figure **3**). Previous studies have reported that TP heterogeneously expressed in different cell lines appears to be N-glycosylated. TP expressed in SF9 cells shows only one band at ~50kDa [10], and that expressed in HEK293 produced two major bands, a 60-66kDa band of presumably fully N-glycosylated receptor and a lower molecular mass non-glycosylated protein of 30–46 kDa [16]. Although heterogeneous glycosylation would not interfere with NMR experiments, it could cause potential problems in crystallization. Previously, it was shown that opsin and β_2_-adrenergic receptor (β_2_-AR) expressed in the HEK293S (GnTI¯)-TetR showed homogenous and restricted N-glycosylation [3,13]. The HEK293S (GnTI¯) cell line is resistant to ricin as a consequence of loss of N-acetylglucosamine transferase 1 (GnTI¯) activity [3]. Therefore, we also constructed HEK293S (GnTI¯)-TetR inducible stable cell lines expressing WT-TP and A160T. WT-TP and A160T expressed in the HEK293S (GnTI¯)-TetR cell line showed homogenous glycosylation and migrate predominantly as a single band with a molecular mass of ~ 37kDa (Figure **3**). We have also evaluated the glycosylation status of WT-TP and A160T expressed in the HEK293S-TetR by pursuing PNGaseF treatment. Treatment with the N-glycosidase resulted in disappearance of the higher molecular weight band, confirming the N-glycosylation status of TP (Figure S1 in File S1).

### Detergent screening

The solubilization of receptors from membranes is a critical step in purification of membrane proteins, thus the detergent used for solubilization is important. Previously, the detergents octyl-β-D-glucoside (OG), n-dodecyl-β-D-maltoside (DM), and 3-[(3-cholamidopropyl) dimethylammonio]-1-propanesulfonate (CHAPS) have been used in WT-TP purification [10,17]. However, we found 80% of WT-TP ligand binding activity was lost when 1% DM was used to solubilize TP from HEK293S-TetR cells (data not shown). This loss of activity was surprising, as 1% DM is routinely used in the purification of GPCRs, including the β_2_-AR [13]. A combination of detergent and cholesterol hemisuccinate (CHS) was used with much success in the purification of a number of GPCRs [18]. To investigate which detergent might be appropriate for obtaining a higher yield at the solubilization step, we carried out a systematic detergent screen. We screened 88 detergents including non-ionic, anionic, cationic and zwitter-ionic detergents. We also carried out solubilization using different percentages of DM and CHS mixtures (Table S1 in File S1). There were clear differences in efficiency of WT-TP solubilization by different detergents as determined by slot blot analysis. However, for those detergents that display a higher level of WT-TP solubilization (C8E6-Anagrade, N-tetradecyl- β-D-maltoside, N-octyl-β-D-maltoside, Anapoe-C12E10, N-dodecyl- β-D-maltoside and Fos-choline-iso-9) there was no statistically significant difference in the functional yield of receptor (Table S1 File S1).

We tried different combinations of DM and CHS and found that addition of 0.2% CHS to 1% DM led to an increase in functional yield of the solubilized WT-TP to 40-45% (Table **1**). This result suggested that membrane cholesterol might be required for TP stability and/or function. However, it remains to be determined whether the modulation of receptor activity observed is due to the direct interaction between cholesterol and TP, or indirect effects caused by the influence of cholesterol on membrane structure or detergent micelle morphology.

**Table 1:** Purification of Thromboxane A2 receptor*.

**Purification step**	**Total protein (mg)**	**Specific Activity (pmol/mg)**	**Activity (pmol)**	**Yield (%)**
Membrane fraction	4.19 ±0.27	16.89 ±1.16	70.37 ±6.18	
Solubilized receptor	3.54 ±0.36	6.71 ±1.28	24.48 ±5.91	39.32 ±3.54
FLAG-affinity purified	0.18 ±0.03	36.46 ±3.57	6.8 ±1.68	10.35 ±0.77

* The receptor was purified using a detergent- lipid mixture containing 1% DM and 0.2% CHS.

### Purification of TP and the A160T CAM

**Figure 4:**
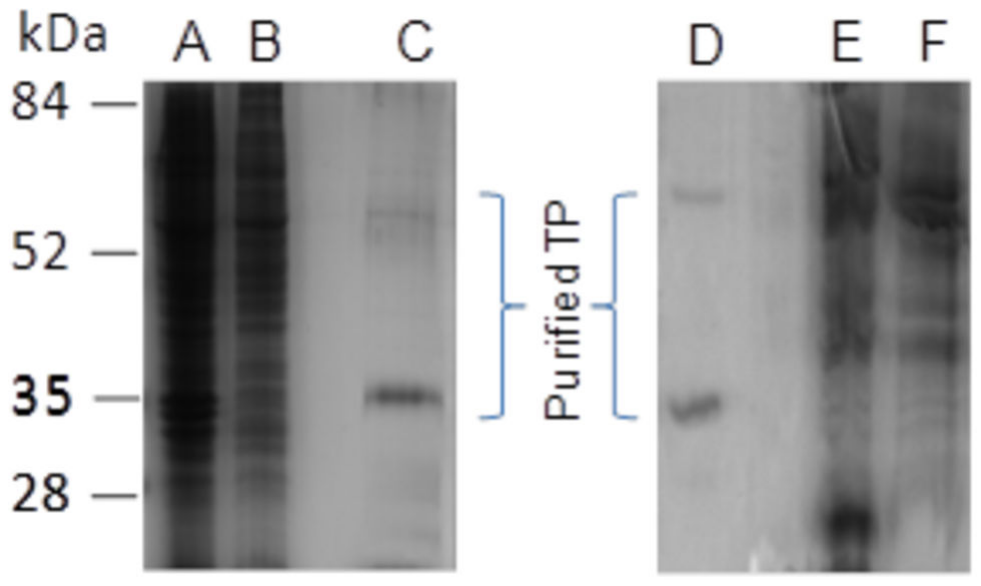
SDS-PAGE (10%) analysis of TP and A160T purification. Membrane preparations (lanes A and E), solubilized (lanes B and F) and FLAG-affinity purified TP and A160T (lanes C and D) from protein expressed in HEK293S-TetR cells. Proteins were detected by Coomassie staining. 5 µg of protein were loaded into all wells. Mobility of molecular weight standards is indicated next to the gel. The receptor produced in HEK293S-TetR was glycosylated and migrated as two bands on the SDS-PAGE with the minor band of ~55 kDa and the major band showing an apparent molecular mass of ~37kDa.

For receptor purification, membranes were prepared from 1.5 x10^7^ HEK293S-TetR cells grown as monolayers in 15 cm dishes. The membranes were solubilized using 1% DM and 0.2% CHS, and the yield of the WT-TP as determined by ligand binding assay was found to be ~40% (Table **1**). In the next step, anti-FLAG M2 agarose was used to purify WT-TP as well as the TP-A160T mutant. The receptors were found to be more than 90% pure, as analyzed by 10% SDS-PAGE (Figure **4**). The receptors produced in HEK293S-TetR was glycosylated, and migrated as two bands with the major band around ~35 kDa and a minor band of ~55 kDa. Previously we have shown by thermal sensitivity assays that the A160T mutant exhibits a 30-40% decrease in stability as compared to WT-TP (9). In line with this observation, we found that the A160T is less stable during the purification, displaying a prominent band at ~25 kDa (Figure **4**, lane E) a proteolysis/degradation product of the C-terminus of A160T, that was detected by the rho-1D4 antibody (data not shown).

The overall recovery of WT-TP obtained after purification using anti-FLAG M2 agarose beads was ~11%. The functional yield of the WT-TP using the single step affinity purification was 45 µg/10^6^ cells. This corresponds to a yield of ~1mg of purified WT-TP from a liter (4.4 x10^7^ cells) of induced HEK293S cells cultured in a bioreactor. We did not carry out ligand binding assays during the purification of the A160T CAM, as it had low affinity for the antagonist [^3^H] SQ 29,548 which is expected for a CAM. The yield of the purified A160T mutant as determined from the total protein eluted after the FLAG affinity purification was comparable to that of WT-TP (Table S2 in File S1).

### Secondary structure analysis of purified TP and A160T mutant

Previously we showed that the A160T mutant exhibits loss of thermal stability [9]. In that study, as only membrane preparations of the mutant and wild type were used, the structural changes could not be characterized. Now, we present the temperature- dependent secondary structure changes of the purified WT-TP and A160T receptors using CD spectropolarimetry. Based on the data from our previous thermal sensitivity assays, we chose to study the proteins at 25°C and 47°C, and at 0 hrs and 3 hrs as time points. Far-UV CD spectropolarimetry confirmed that both the WT-TP and A160T mutant are predominantly α-helical. The spectra were analyzed using the K2D3 algorithm, which predicted an α-helix content of ~70% for both the WT-TP and A160T mutant.

**Figure 5:**
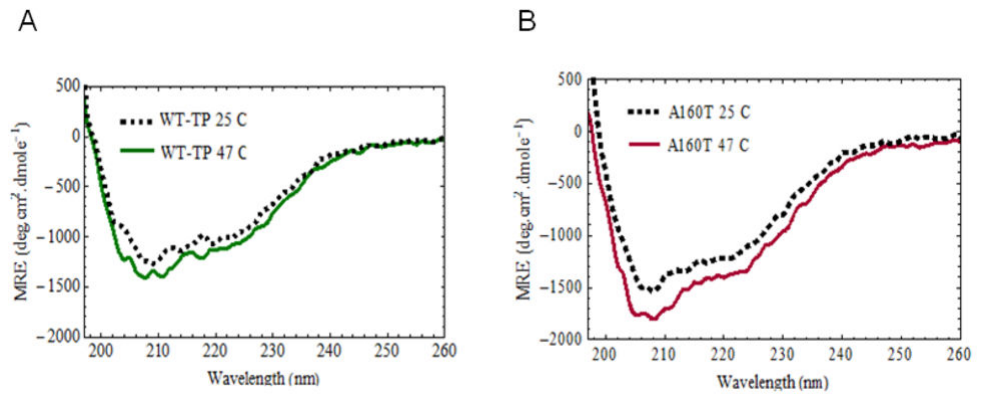
Circular dichroism spectra of purified TP and A160T mutant at different temperatures. The spectra show typical α-helical profiles with minima at 208 nm and 222 nm. These results suggest that WT-TP (panel A) and A160T (Panel B) are folded correctly. The A160T mutant is thermally more sensitive and shows a subtle loss of secondary structure compared to WT-TP.

The results in Figure 5 suggest that the A160T mutant secondary structure is less stable than the WT. Heating for 3 hr has only a very small effect on the WT-TP and causes a measurably larger loss of secondary structure in the mutant (Figure **5**). This suggests that the loss of activity or thermal sensitivity that was previously observed for the A160T, is not owing to large unfolding of the protein but rather to a more subtle effect. Our results suggest that very little change occurs between 0 hrs and 3 hrs at room temperature for both the A160T mutant and the WT-TP (data not shown). For the mutant, it appears that no change occurs over the 3 hr incubation at 47 °C (Figure **5B**). This suggests that all the change in conformation took place during the 5 minutes that we allowed for the sample to warm up from 25 °C to 47 °C. In contrast, for the WT-TP the difference between 0 hr and 3 hr spectra suggests there is very little change in the first 5 minutes of heating (data not shown).

Recently, the purification of glycosylated WT-TP in milligram amounts using a baculovirus expression system was reported [10]. However, none of the studies on the prostanoid receptors reported the ability to express and purify a homogenously glycosylated receptor, or a CAM at high-levels. The effect of various detergents on prostanoid receptor function or stability was also not tested.

In conclusion, our studies revealed that using the HEK293S-TetR-inducible system both WT-TP and the A160T CAM are expressed at up to 4-fold higher levels, and they showed homogenous glycosylation when expressed in the HEK293S (GnTI¯)-TetR cell line. The yield of the functional receptor obtained from the one-step purification now makes it feasible to purify WT-TP and CAMs in milligram amounts from mammalian cells. Furthermore, additional approaches can be used to improve the stability and yield of TP and mutants. These include addition of antagonist to cultures expressing TP or agonist to cultures expressing the CAMs, and inclusion of ligands during the purification process might also increase stability of the protein. Scale up of the expression using suspension cultures in a bioreactor using established techniques should allow production of TP, and importantly the A160T CAM at levels suitable for the application of high-resolution biophysical studies, such as NMR spectroscopy. This is the first study to report on the successful high-level expression, purification and biophysical characterization of a naturally occurring, diffusible ligand activated GPCR variant that exhibits constitutive activity.

## Materials and Methods

### Materials

Anti-FLAG M2 affinity gel (Cat # A2220), FLAG peptide (Cat # F3290), and FLAG antibody were from Sigma. All of the lipids and detergents, including n-dodecyl-β-D-maltoside were purchased from Anatrace. Common chemicals and reagents were purchased from either Sigma or Fisher. Restriction enzymes were from NEB, and cell culture supplies were purchased from Invitrogen. The radiolabeled ligand [^3^H] SQ 29,548, was purchased from PerkinElmer (NET 936), and cold SQ 29,548 was obtained from Cayman Chemicals (Cat # 19025).

Buffers used were as follows: PBS buffer, 137 mM NaCl, 2.7 mM KCl, 1.8 mM KH_2_PO_4_, 10 mM Na_2_HPO_4_ (pH7.4); Buffer A (lysis buffer), 10 mM Tris-HCl, pH 7.4, containing protease inhibitors (1mM EDTA, 10µg/ml benzamidine, 10 µg/ml leupeptin, 20 µg/ml soybean trypsin inhibitor, and 0.2mM phenylmethylsufonyl fluoride); Buffer B (storage buffer), 50 mM Tris-HCl, pH 7.4, 12.5 MgCl_2_, containing protease inhibitors as in Buffer A; Buffer C (binding buffer), 75 mM Tris-HCl, pH 7.4, 12.5 mM MgCl_2_, containing protease inhibitors as in Buffer A; Buffer D (solubilization buffer) 50 mM Tris-HCl, pH 7.4, 150 mM NaCl, 10% glycerol, 1 mM EDTA, containing protease inhibitors as in Buffer A; Buffer E (Elution buffer) 50 mM Tris-HCl, pH 7.4, 150 mM NaCl.

### Construction of tetracycline-inducible HEK293S stable cell lines expressing TP and A160T

The genes FLAG-TP-1D4 and FLAG-TP-A160T-1D4 in plasmid pUC57 and codon-optimized for expression in mammalian cells were synthesized commercially (GenScript Inc, USA). These two genes in pUC57 and pACMVtetO were digested with restriction enzymes Kpn1 and NotI for 2 h at 37 °C. After removal of the enzyme using Qiagen gel purification kit, the TP fragment was ligated into the plasmid pACMVtetO, and transformed into competent *E. coli* DH5α. The transformants were screened for the presence of the 1.2 kb TP and A160T genes following digestion with Kpn1 and NotI. Their identity was confirmed by DNA sequencing. The plasmids with the correct gene sequence were then transfected into HEK293S-TetR and HEK293S-TetR (GnTI¯) cells using lipofectamine 2000. The expression and selection were carried out as described previously [13]. The expression of the clones using slot blot was detected using the anti FLAG-antibody and visualized by chemiluminescence (ECL, Amersham).

### Systematic detergent screening

Detergent screening was carried out using the solution master detergent kit from Anatrace containing 88 detergents. WT-TP was solubilized in lysis buffer containing various detergents for 1 h at 4 °C, and samples were analyzed on a dot blot. The ability of each detergent to solubilize WT-TP was quantified using ImageJ software.

### Purification of WT-TP and A160T

Cell pellets from two dishes (15 cm each) were resuspended using 100 ml of Buffer A. The suspension was homogenized using a dounce homogenizer (20 strokes), and centrifuged at 48,000 xg for 30 min. After weighing the membrane pellet, each gram of membrane pellet were suspended in 10 ml buffer D containing 1% DM and 0.2% CHS using a dounce homogenizer (20 strokes). The suspension was mixed by nutation at 4 °C for 1 h, and centrifuged at 48,000 xg for 30 min to remove any insoluble particulate material. Solubilized TP or A160T were incubated with FLAG-resin in batch mode (binding capacity of FLAG resin is 0.6 mg/ml) with slow nutation for 2 h at 4°C. The receptor bound resin was then collected by centrifugation at 1500 xg and washed with Buffer D containing 0.05% DM and 0.01% CHS until the absorbance of the wash at 280 nm was < 0.01. Elution was carried out with Buffer E containing 0.05% DM, 0.01% CHS and 0.1 mM FLAG peptide. The fractions obtained were assayed for receptor binding using [^3^H] SQ 29,548 and/or the protein concentration was determined by a Biorad DC protein assay. Radioligand binding assays were as described previously [9,14].

### Immunoblot analysis

One to five micrograms of the protein sample were resolved by using a 10% gel by SDS-PAGE. The protein was then transferred from the gels onto a nitrocellulose membrane by electroblotting. The WT-TP and the A160T mutant receptor were visualized by immunodetection with the anti FLAG-antibody or rho-1D4 antibody.

### Circular dichroism (CD) spectropolarimetry

CD spectra were recorded on a JASCO J-810 spectropolarimeter at the indicated temperatures over the wavelength range of 190 nm to 260 nm with a step size of 1nm. Spectra were collected with a cylindrical quartz sample cell with a path length of 0.05 cm. Spectra of purified receptors were baseline corrected by subtracting the spectra of buffer solutions. The intensity and wavelength of the spectropolarimeter were calibrated using solutions of d-10-camphorsulfonic acid. Mean Residue Ellipticities (10^-3^ deg.cm^2^. dmole^-1^) were calculated using the equation: [Ɵ] M = (Mr) (Ɵ)/(10) (l) (c) (n), where Mr is 39,212.8 grams per mole for WT-TP and 39,242.8 grams per mole for the A160T mutant, l is the cell path length in cm, Ɵ is the measured ellipticity in millidegrees, c is the protein concentration in g/L, and n = 359. CD spectra were deconvoluted using the K2D3 algorithm (http://www.ogic.ca/projects/k2d3/) [19]. The CD spectra were processed using Wolfram Mathematica 9 (Wolfram Research, Inc., IL, USA).

## Supporting Information

File S1 **Figure S1**. Immunoblot analysis of TP and A160T variant digested with PNGaseF and detected using the monoclonal FLAG antibody. TP and the A160T variant were expressed in HEK293S (GnTI-) cell line with restricted and homogeneous N-glycosylation (lanes A and D). TP and the A160T variant were expressed in the HEK293S-TetR stable cell line (lanes B and E). TP and the A160T variant expressed in the HEK293S-TetR stable cell line were digested with PNGase F (New England Biolabs) (lanes C and F). TP and the A160T variant were treated with 0.5% SDS and 40 mM DTT at 100 °C for 10 min and then added 50 mM Na _3_PO_4_ buffer (pH 7.5), 1% NP-40 and 2 µl PNGase F, and incubated at 37 °C for 1 h. Equal amount (10 µg) of protein were loaded in all wells. The size of the molecular weight standards is indicated next to the gel. **Table S1**. Detergent screen for solubilization of FLAG-TP expressed in HEK293S-TetR stable cell line. **Table S2**. Purification of the CAM A160T from HEK293S-TetR and HEK293S (GnTI^-^) -TetR stable cell lines.
(DOC)
