# Supplementary material for: High-Level Expression, Purification and Characterization of a Constitutively Active Thromboxane A2 Receptor Polymorphic Variant
Source: PLoS One. 2013 Sep 23;8(9):e76481. doi: 10.1371/journal.pone.0076481 (PMC3781061; doi:10.1371/journal.pone.0076481)
Supplement: File S1 — Figure S1. Immunoblot analysis of TP and A160T variant digested with PNGaseF and detected using the monoclonal FLAG antibody. TP and the A160T variant were expressed in HEK293S (GnTI-) cell line with restricted and homogeneous N-glycosylation (lanes A and D). TP and the A160T variant were expressed in the HEK293S-TetR stable cell line (lanes B and E). TP and the A160T variant expressed in the HEK293S-TetR stable cell line were digested with PNGase F (New England Biolabs) (lanes C and F). TP and the A160T variant were treated with 0.5% SDS and 40 mM DTT at 100 °C for 10 min and then added 50 mM Na 3PO4 buffer (pH 7.5), 1% NP-40 and 2 µl PNGase F, and incubated at 37 °C for 1 h. Equal amount (10 µg) of protein were loaded in all wells. The size of the molecular weight standards is indicated next to the gel. Table S1. Detergent screen for solubilization of FLAG-TP expressed in HEK293S-TetR stable cell line. Table S2. Purification of the CAM A160T from HEK293S-TetR and HEK293S (GnTI-) -TetR stable cell lines. (DOC) [file pone.0076481.s001.doc]

**SUPPORTING INFORMATION**

**
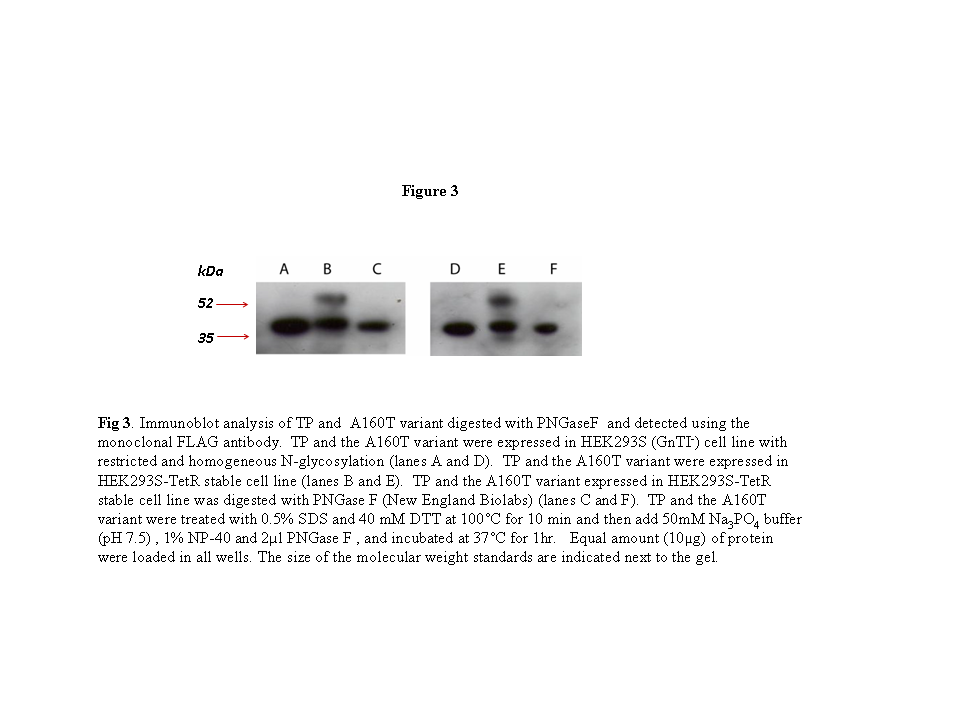
**

**Figure S1**. Immunoblot analysis of TP and A160T variant digested with PNGaseF and detected using the monoclonal FLAG antibody. TP and the A160T variant were expressed in HEK293S (GnTI-) cell line with restricted and homogeneous N-glycosylation (lanes A and D). TP and the A160T variant were expressed in the HEK293S-TetR stable cell line (lanes B and E). TP and the A160T variant expressed in the HEK293S-TetR stable cell line were digested with PNGase F (New England Biolabs) (lanes C and F). TP and the A160T variant were treated with 0.5% SDS and 40 mM DTT at 100 °C for 10 min and then add 50 mM Na3PO4 buffer (pH 7.5), 1% NP-40 and 2µl PNGase F, and incubated at 37 °C for 1 h. Equal amount (10 µg) of protein were loaded in all wells. The size of the molecular weight standards is indicated next to the gel.

**Table S1. Detergent screen for solubilization of FLAG-TP expressed in HEK293S-TetR stable cell line.**

**Table S2. Purification of the CAM A160T from HEK293S-TetR and HEK293S (GnTI^-^**) -TetR stable cell lines*

| **Purification step** | **HEK293S-TetR Total protein (mg)** | **HEK293S (GnTI^-^)-TetR**  **Total protein (mg)** |
| --- | --- | --- |
| Membrane fraction | 6.21 | 5.27 |
| Solubilized receptor | 5.82 | 4.84 |
| FLAG-affinity  Purified receptor | 0.31 | 0.35 |

*Radioligand assays were not performed as the CAM A160T displayed weak affinity for the antagonist radiolabel [^3^H] SQ 29,548. Hence, the amount of receptor obtained in terms of protein concentration is displayed at each purification step.
